# Effects of Resistance Exercise on Mental Health in Older Chinese Americans: A Randomized Controlled Trial

**DOI:** 10.1093/geroni/igaa057.605

**Published:** 2020-12-16

**Authors:** Mei-Lan Chen, Elisabeth Burgess, Ying-Yu Chao, Douglas Gardenhire, Ruiyan Luo

**Affiliations:** 1 Georgia State University, Atlanta, Georgia, United States; 2 Rutgers School of Nursing, Newark, New Jersey, United States

## Abstract

Regular exercise has shown to be potentially beneficial for improving mental health in older adults. However, few studies evaluated the effect of resistance exercise on psychological well-being in older Chinese Americans. The purpose of this two-arm randomized controlled trial (RCT) was to test the effects of resistance exercise training on stress, depression, and social engagement in community-dwelling older Chinese Americans. A total of 30 older adults (mean age 77.9 ± 5.0 years) were randomly assigned into the resistance exercise intervention group (n = 15) or the wait-list control group (n = 15). The resistance training intervention includes 50-min group exercise session twice weekly for 12 weeks. Participants’ perceived stress, depressive symptoms, and social engagement were measured at baseline and 12 weeks follow-up. Descriptive statistics and t tests were performed for data analysis. The results revealed that the resistance exercise intervention group had significant improvements in perceived stress, depressive symptoms, and social engagement after receiving the 12-week intervention. At baseline, there were no significant differences between the intervention and the control groups on perceived stress, depressive symptoms, and social engagement. However, older adults received resistance exercise training had greater improvements in stress levels, depressive symptoms, and social engagement than their control counterparts at 12 weeks follow-up. The findings suggest resistance exercise has positive effects on psychosocial well-being for older adults. Further larger RCTs are needed to assess long-term effects of the resistance exercise intervention.

